# Heat shock protein 90: translation from cancer to Alzheimer's disease treatment?

**DOI:** 10.1186/1471-2202-9-S2-S7

**Published:** 2008-12-03

**Authors:** Wenjie Luo, Anna Rodina, Gabriela Chiosis

**Affiliations:** 1 Laboratory of Molecular and Cellular Neuroscience, The Rockefeller University and Fisher Foundation for Alzheimer's Disease, New York, NY 10021, USA; 2 Department of Medicine and Program in Molecular Pharmacology and Chemistry, Memorial Sloan-Kettering Cancer Center, New York, NY 10021, USA

## Abstract

Both malignant transformation and neurodegeneration, as it occurs in Alzheimer's disease, are complex and lengthy multistep processes characterized by abnormal expression, post-translational modification, and processing of certain proteins. To maintain and allow the accumulation of these dysregulated processes, and to facilitate the step-wise evolution of the disease phenotype, cells must co-opt a compensatory regulatory mechanism. In cancer, this role has been attributed to heat shock protein 90 (Hsp90), a molecular chaperone that maintains the functional conformation of multiple proteins involved in cell-specific oncogenic processes. In this sense, at the phenotypic level, Hsp90 appears to serve as a biochemical buffer for the numerous cancer-specific lesions that are characteristic of diverse tumors. The current review proposes a similar role for Hsp90 in neurodegeneration. It will present experimentally demonstrated, but also hypothetical, roles that suggest Hsp90 can act as a regulator of pathogenic changes that lead to the neurodegenerative phenotype in Alzheimer's disease.

## Background

Neurodegenerative diseases, including Alzheimer's disease (AD), are characterized by the progressive dysfunction of normal physiological cellular events. Whereas the outcome of pathogenic changes in the brain is manifested in a complex set of hallmarks that are different when compared to cancer, the passage into neurodegenerative disease has many similarities to malignant transformation. In this review, we will present recent findings suggesting that heat shock protein 90 (Hsp90) may play a role in maintaining pathogenic changes that lead to neurodegenerative diseases. We will also speculate on yet unexplored putative roles of this chaperone in the particular case of AD.

## Cancer and Hsp90

Transformation of normal cells into malignant cells is a multistep process requiring the accumulation of a number of genetic alterations influencing key regulatory processes. In this regard, many types of cancers are diagnosed in the human population with an age-dependent incidence that implicates several events that take the cell from premalignant states into invasive cancers [1]. Dysregulations may occur in a multitude of pathways and be evidenced through protein mutation, misexpression, or misproccessing, leading to altered functions that confer a pathogenic cell phenotype. While at the cellular level these dysregulations are advantageous in cancer, and may lead to increased survival, at the molecular level, these changes take place at a cost to local energetic stability. To regain a pseudo-stable state, cells co-opt chaperones, for example, Hsp90, to bind aberrant proteins involved in the dysregulated processes with high-affinity and maintain them in a functional conformation [2-6]. These interactions buffer the local molecular instability and allow for the accumulation of aberrant proteins that ultimately leads to the blossoming of disease. Thus, following dysregulation in the abundance, stability or activity of a given protein, cell survival can become critically dependent on the association of client proteins of non-native stability with Hsp90.

In cancer, Hsp90 and associated co-chaperones were found to assist in the correct conformational folding of transformation-specific 'client proteins' without significantly binding to, or influencing the folding of, 'normal' protein counterparts; many of these client proteins are signal-transduction regulators of cell growth, differentiation, the DNA damage response, and cell survival [2-6]. Small molecule inhibitors of Hsp90 disturb its association with aberrant proteins and stimulate their degradation, a process initiated by recruitment of E3-ligases and mediated by the proteasome [2-7].

Historically, v-Src kinase was the first oncoprotein shown to display unusually stable interactions with Hsp90 and associated chaperones [8]. In contrast, non-oncogenic c-Src requires only limited assistance from the Hsp90 machinery for its maturation and cellular function. Similarly, stable expression of the mutant, but not wild-type, p53 conformation required tight association of the p53 protein with Hsp90 [9]. In the chronic myelogenous leukemia cell line K562, transformation is driven by the aberrant fusion of the genes *bcr *and *abl*, leading to the production of a constitutively active kinase, Bcr-Abl. Hsp90, which is minimally required for the stabilization of Abl itself, becomes closely associated with Bcr-Abl and maintains the kinase's functionality in this dysregulated state [10,11]. Nucleophosmin-anaplastic lymphoma kinase, found in lymphomas, is another recognized tumor-specific client of Hsp90 [12], as is mutated Flt3, a kinase involved in driving transformation in acute myeloid leukemias [13]. Steroid-hormone receptors in breast and prostate cancers have an important role in the malignant behavior of these tumors. They too are examples of tumor-specific clients where oncogenic activity can be disrupted by Hsp90 inhibitors [14,15]. Epidermal growth factor receptor harboring kinase-activating mutations that are involved in the transformation of non-small cell lung cancers also associates with Hsp90. An inhibitor of Hsp90 triggers the rapid degradation of these kinases without affecting wild-type epidermal growth factor receptor [16]. Zeta-chain-associated protein kinase 70 (ZAP-70), expressed in patients with aggressive chronic lymphocytic leukemia (CLL) and required for cell survival and signaling in CLL, behaves as an Hsp90 client protein only in CLL cells [17]. Examples may be extended to numerous additional transformed cell types but, in sum, multiple proteins involved in cell-specific oncogenic processes have been shown to be tightly regulated by the binding of Hsp90 and undergo selective degradation following treatment with an Hsp90 inhibitor. In this sense, at the phenotypic level, Hsp90 seems to serve as a biochemical buffer for the numerous cancer-specific lesions that are characteristic of diverse tumors.

In an effort to refine the many characteristics that are required for the development of the fully malignant phenotype, Hanahan and Weinberg proposed six essential phenotypic traits, referred to as the 'six hallmarks' of a cancer cell [1]. Common to these hallmark traits is Hsp90, a protein that has the capacity to regulate key elements of each of these processes, suggesting that the chaperone is an indispensable controller of multiple proteins regulating these cancer hallmarks [1-6,18].

In summary, malignant cells co-opt Hsp90 to maintain their viability under the pressure of aberrant proteins and, therefore, allow malignant transformation and the facilitation of disease progression. Hsp90 inhibition therefore offers the potential of accomplishing what most targeted anticancer therapies do not: the simultaneous disruption of multiple signaling events critical to all recognized cancer hallmarks. In consequence, the unique biological role of Hsp90 in cancer cells has suggested that its inhibition could be an answer to the challenge imposed on therapy by the heterogeneity and adaptability of cancer cells, and represent a singular therapeutic modality against a large array of tumors.

## Neurodegenerative diseases and Hsp90

For neurodegenerative disorders associated with protein aggregation, the view on Hsp90 has been limited to its regulation of heat shock response [19-21]. Inhibition of Hsp90 activates heat shock factor 1 (HSF1) to induce the production of the chaperones Hsp70 and Hsp40, which promote disaggregation and protein degradation. It is suggested that under non-stressed conditions, Hsp90 binds to HSF1 and maintains the transcription factor in a monomeric state [22]. Inhibition of Hsp90 releases HSF1 from the Hsp90 complex, leading to its trimerization, activation and translocation to the nucleus where it initiates a heat shock response. However, recent evidence suggests an additional role for Hsp90 in neurodegenerative diseases [23-26]. Based on the large body of evidence on the ubiquitous 'transformation buffering' potential of Hsp90 in cancer, it is intuitive to suggest analogous roles for Hsp90 in neurodegeneration.

### Spinal and bulbar muscular atrophy

Spinal and bulbar muscular atrophy (SBMA) is an inherited motor neuron disease caused by the expansion of a polyglutamine tract within the androgen receptor (AR) [27]. The pathological features of SBMA are motor neuron loss in the spinal cord and brainstem, diffuse nuclear accumulation, and nuclear inclusions of the mutant AR in the residual motor neurons and certain visceral organs. Waza *et al. *[23] recently demonstrated that mutant AR, as present in SBMA, is an Hsp90 client protein that forms a molecular complex with the chaperone. This complex is required to maintain the functional stability of the mutant AR. Addition of 17-allylamino-17-demethoxy geldanamycin (17AAG), a small molecule Hsp90 inhibitor currently in phase II evaluation in patients with advanced cancers, to both cells and transgenic mice led to a preferential degradation of mutant AR compared to wild-type. These effects were a result of increased dependency of the mutant AR, compared to its normal counterpart, on Hsp90 for stability, and not due to an induction of Hsp70 and Hsp40. In a SBMA transgenic mouse model, 17AAG ameliorated motor impairments without detectable toxicity and reduced the amounts of monomeric and aggregated mutant AR. Similar findings were reported by Thomas *et al. *[24], who found that Hsp90 inhibition blocked the aggregation of the expanded glutamine androgen receptor (AR112Q) in HSF1(-/-) mouse embryonic fibroblasts where the Hsp70 and Hsp40 chaperones were not induced.

### Tauopathies

Tauopathies are neurodegenerative diseases characterized by tau protein abnormalities. In these diseases, transformation is characterized by abnormalities in the tau protein that lead to the accumulation of hyperphosphorylated and aggregated tau [28,29]. It has been suggested that AD and frontotemporal dementia are linked in a genetic spectrum of presenile degenerative brain disorders in which tau is one of the important players [30]. In AD, tau hyperphosphorylation is suggested to be a pathogenic process caused by aberrant activation of several kinases, in particular cyclin-dependent protein kinase (cdk)5 and glycogen synthase kinase (GSK)3β, leading to phosphorylation of tau at pathogenic sites. Hyperphosphorylated tau in AD is believed to misfold, undergoing net dissociation from microtubules, and form toxic aggregates [31,32]. In a cluster of tauopathies termed 'frontotemporal dementia and parkinsonism linked to chromosome 17 (FTDP-17)', pathology is caused by several mutations in human tau isoforms on chromosome 17, which result in, and are characterized by, the accumulation of aggregated tau, similar to that in AD [33,34]. Over 20 pathogenic mutations have been identified but P301L is the most common among tauopathies. Luo *et al. *[25] recently presented evidence that, in a particular case of tauopathy, the stability of both p35, a neuronal protein that may activate cdk5 through complex formation leading to aberrant tau phosphorylation, and the P301L mutant, but not wild-type tau, are maintained by Hsp90. These proteins form a molecular complex with the chaperone that is necessary to regulate their function and stability. Inhibition of Hsp90 in both cellular and mouse models of tauopathies by Hsp90 inhibitors of the purine-scaffold class [35] led to the reduction of the aberrant activity of these proteins and resulted in a decrease of aggregated tau. Complementary results were generated by Dickey *et al*. [26], who demonstrated that inhibition of Hsp90 by a similar small molecule led to a decrease in phosphorylated tau levels independent of HSF1 activation. This reduction occurred selectively in the aberrant phosphorylated tau species, leaving normal tau largely unaffected.

Luo *et al. *[25] and Dickey *et al. *[26] have also shown that the Hsp90 onco-clients Akt and Raf-1 were mainly unaltered by Hsp90 inhibitors. These important findings suggest that 'tight' regulation of aberrant proteins by Hsp90 is driven by the pathogenic event itself and, therefore, manifested in a pathogenic-specific manner. Dickey *et al. *[26] further demonstrated that the Hsp90 complex in affected areas of AD brain has a significantly higher binding affinity (approximately 1,000-fold) for small molecule inhibitors than Hsp90 derived from unaffected brain tissue from the same patients or from controls.

Collectively, these findings suggest that a neuron undergoing a degenerative process may co-opt Hsp90 in a fashion similar to an epithelial cell undergoing malignant transformation. In doing so, it maintains the functional stability of proteins of aberrant capacity, allowing and sustaining their accumulation as toxic aggregates, thus providing a common principle that governs the two diseases. The process is ill-fated for neurons, ultimately resulting in the loss of disease-specific classes, unlike the increased cellular survival seen in cancer.

## Hsp90: putative roles in Alzheimer's disease

AD, the most common neurodegenerative dementia in the elderly, affects cognition, behavior and functioning. It is a heterogeneous disease in which the pathogenic transformation is probably driven by a multitude of aberrant events, and as in cancer, no two patients present an identical disease. The major hallmarks accepted for AD include: amyloid deposition composed of β-amyloid peptide (Aβ); intracellular neurofibrillary tangles composed of abnormally phosphorylated forms of the protein tau; prominent neuroinflammation of nearby glial cells; and synaptic loss and specific neuronal death.

Several hypotheses on the basis of the disease have emerged, but it is yet unclear whether these are causative events or neuronal pathways prone to being hijacked by pathogenic elements. Irrespective of the initiator factor, a large body of evidence suggests aberrant activation of essential kinases in these pathways is associated with AD progression. Gradually, dysregulated kinase activities might contribute in an age-dependent manner to amyloid generation and deposition, tau hyperphosphorylation and tangle formation, neuroinflammation, and ultimately to neuronal death.

### Amyloid generation and deposition

**Figure 1:**
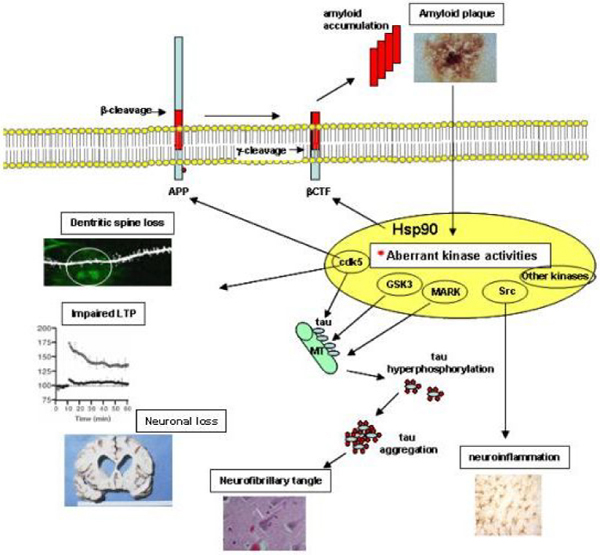
Proposed model for the regulatory roles played by Hsp90 in AD progression. Hsp90 can promote AD by facilitating the activities of protein kinases that cause the pathological features of AD. LTP, long-term potentiation; CTF, carboxyl-terminal fragment – cleavage product of APP (amyloid precursor protein); MT, microtubule.

Aβ, the major component of the extracellular amyloid deposits, is believed to be the upstream causative factor in the AD pathological cascade generated by the sequential proteolytic cleavage of the amyloid precursor protein (APP), as summarized in Figure 1[34]. The length of Aβ peptides generated can vary. Among the most common forms, Aβ42 is far more prone to aggregation than the more abundant Aβ40 [35]. Under physiological conditions, the steady state concentrations and ratio of Aβ40 and Aβ42 are balanced, and thus no pathogenic deposits form. Although the mechanisms for pathogenic deposition of Aβ are still unclear, it is speculated that several factors, including familial Alzheimer's mutations, genetic risk factors (for example, apolipoprotein E), environmental stress, and decreased Aβ clearance capability, may disrupt the balance and result in accumulation of amyloid peptides and promote aggregation. The build-up of Aβ may initiate multistep pathogenic events, including disruption of neuronal homeostasis, and the aberrant activation of kinases. These alterations ultimately lead to neurofibrillary tangle formation, prominent neuroinflammation, and neurodegeneration [34].

Among the Aβ-induced aberrant kinases, cdk5 has been implicated in AD pathology. In human AD brains, there is a significant, specific elevation in cdk5 activity compared with age-matched controls [36,37]. Normally, activation of cdk5 is regulated by association with its cofactor, p35 [38]. It is also believed that elevated cdk5 activity in AD may be induced by p25, a more stable cleavage form of p35 [39,40]. In this case, Aβ accumulation activates intracellular calcium signaling and induces the pathogenic production of p25 via a calpain-dependent cleavage of p35. Upon binding to cdk5, p25 causes mislocalization and prolonged activation of cdk5. The p25-activated cdk5 complex alters substrate specificity, preferentially phosphorylating tau and APP [41-44]. Aberrant kinase activities also regulate Aβ generation. Cdk5 phosphorylation of APP on Thr668 can induce a conformational change in APP, altering its intracellular trafficking to facilitate β-secretase cleavage and Aβ generation [45]. A positive feedback loop could therefore exist between cdk5 and Aβ, where accumulation of Aβ induces aberrant cdk5 activation that in turn further stimulates Aβ generation, ultimately triggering a cascade of pathogenic events (Figure 1) [46]. Similarly, Aβ can activate GSK3β, leading to increased Aβ production [47]. Casein kinase 1 (CK1) is also implicated in regulating Aβ-generation. Constitutive overexpression of active CK1ε, one of the CK1 isoforms expressed in brain, can generate an increase in Aβ peptide production [48].

Another theory, complementary to the amyloid hypothesis and which also invokes signal transduction, proposes that APP and presenilins may modulate an as yet unknown cell signal, the disruption of which may induce cell-cycle abnormalities, amyloid formation, neuronal death, and eventually dementia [49].

### Tau pathology

One of the major pathologies of AD, hyperphosphorylation of the microtubule-binding protein tau and formation of intracellular neurofibrillary tangles, is suggested to result from abnormal activation of essential kinases (Figure 1) [28-30,32]. The hyperphosphorylation of tau at pathogenic sites causes its detachment from microtubules, perturbing normal microtubule function. Hyperphosphorylated species form paired helical filaments that easily aggregate and may ultimately act as physical barriers to axonal transport, impairing synaptic transmission [50]. Tau hyperphosphorylation may be caused by various events, among which up-regulated or aberrant activation of tau kinases (for example, cdk5, GSK3β, mitogen-activated protein kinases (MAPK), calcium/calmodulin-dependent kinase II (caMK-II), and the microtubule-affinity-regulating kinase (MARK)) are believed to play important roles (Figure 1) [51,52].

### Neuroinflammation

Neuroinflammation, another prominent feature of AD, is caused by microglia and astrocytes (Figure 1). These cells are activated in AD brain, as well as in AD transgenic mice [53-55]. Recent studies implicate several signaling pathways of neuroinflammation in AD. Microglial activation is suggested to result from Aβ binding and activation of cell surface immune and adhesion molecules, for example, CD45, CD40, CD36 and integrins. Subsequently, members of the Src family of tyrosine kinases, including Fyn, Lyn and Syk, are recruited to activate the ERK and MAPK pathways, inducing proinflammatory gene expression, and leading to the production of cytokines and chemokines. This chain of events leads to further microglial activation, astrogliosis, secretion of proinflammatory molecules, Aβ generation, and tau phosphorylation, thus perpetuating the cascade [56]. Interestingly, recent studies using AD-transgenic mice indicate that inflammation also contributes to tau pathology by a cdk5/p25-mediated pathway [57].

### Synaptic and neuronal loss

Synaptic loss is an early event in AD and the best correlate of cognitive dysfunction [58]. Aberrant cdk5 activity in AD can also cause dendritic spine loss and disrupt synaptic activity. In this regard, phosphorylation of WAVE1 by cdk5 inhibits WAVE1's activity in spine development and results in a decrease in mature dendritic spines [59]. In a transgenic model, prolonged p25 production and cdk5 activation caused severe cognitive deficits that were accompanied by synaptic and neuronal loss, and impaired long-term potentiation [60]. Neuronal death in AD is also linked to aberrant kinase activity and several signaling pathways that cascade into cell death have been identified [61-64]. In a new twist, cdk5 interacts with p53 and increases its stability through post-translational regulation, leading to the accumulation of p53 (particularly in the nucleus), and on to neuronal death [65]. With respect to the 'cell cycle-like reactivation' hypothesis, pathological kinase activity has again been identified [65-67]. Terminally differentiated neurons remain in G0 phase and display, compared to proliferating cells, an opposite regulation pattern of cell cycle markers where most of the key activators and inhibitors are down- and up-regulated, respectively. Experimental attempts to force terminally differentiated neurons to divide ultimately leads to their death. Conversely, cell cycle blockade in experimental models of neuronal death is able to rescue neurons, suggesting that cell cycle dysregulation is among the mechanisms governing neuronal death. For the p25/cdk5 kinase complex, a role in this pathological process has been suggested through retinoblastoma protein phosphorylation and derepression of E2F-responsive genes [68].

Collectively, these findings suggest aberrant kinase activation is a critical step in the cascade of detrimental events that both initiate and permit the development of the pathogenic events in AD. To tolerate the accumulation of these dysregulated processes, and allow the blossoming of the disease phenotype, their functional stability likely requires a 'buffering' mechanism, as offered by Hsp90 in malignant transformation. In Figure 1, we present our view on the putative roles Hsp90 may play in AD. This model suggests Hsp90 as a master regulator of pathogenic events leading to AD. We hypothesize that accumulated amyloid may trigger a cascade of cellular changes, including altered kinase activities. These aberrant kinase activities develop Hsp90-dependency and promote disease progression. The outcomes of 'Hsp90-sheltered' aberrantly activated proteins are tau hyperphosphorylation, synaptic deficits, and neuroinflammation, salient determinants of the pathological changes of AD that lead to amyloid deposition, tangle formation, synaptic dysfunction, and neuronal death.

## Conclusion and significance

The bulk of current AD research is focused on possible interventions along the amyloid pathways. However, this focused approach may not ameliorate outcomes due to abnormal tau phosphorylation. In addition, AD is a complex and heterogeneous disease, with a diversity of risk factors and a multitude of symptoms. In the post-genomic era, identification of novel molecular targets for AD may offer the theoretical promise of great specificity coupled with reduced systemic toxicity, but this highly focused targeting approach faces the potential peril of being unable to deal successfully with a complex disease, such as AD. We speculate that targeting Hsp90, part of the cellular machinery that allows the accumulation and progression of dysregulated events in AD, could provide a more comprehensive approach towards treatment. The model proposes a multifaceted use for Hsp90 inhibitors and presents a view whereby targeting one protein, Hsp90, may ameliorate several aspects of the disease. Hsp90 inhibition may restore a multitude of damaged signaling networks in the diseased brain by alleviating aberrant phosphorylation and reducing protein misprocessing. The ability of Hsp90 inhibitors to simultaneously affect multiple transforming molecules and pathways is a unique and therapeutically attractive feature of targeting this chaperone [69]. These findings suggest that Hsp90 inhibitors might provide a broader, more effective anti-neurodegenerative therapy than molecules targeting single signaling molecules that are the focus of most current drug discovery efforts. Moreover, the apparent increased requirement for Hsp90 activity in cancer suggests the real potential of an exploitable therapeutic index for this approach in neurodegenerative diseases.

Although a target still in its infancy in AD, Hsp90 has recently become the focus of several research efforts. It may take several years until its promise in AD treatment may come to fruition, and it will likely require concerted efforts that entail a better understanding of the biology of Hsp90 in AD, but also the development of small molecule Hsp90 inhibitors better suited for central nervous system use.

## List of abbreviations used

17AAG: 17-allylamino-17-demethoxy geldanamycin; Aβ: β-amyloid; AD: Alzheimer's disease; APP: amyloid precursor protein; AR: androgen receptor; cdk: cyclin-dependent protein kinase; CK: casein kinase; CLL: chronic lymphocytic leukemia; GSK: glycogen synthase kinase; HSF1: heat shock factor 1; Hsp: heat shock protein; MAPK: mitogen-activated protein kinase; SBMA: spinal and bulbar muscular atrophy.

## Competing interests

The authors declare that they have no competing interests.
